# Soil biodiversity and network complexity jointly drive soil multifunctionality in an open cast coal mine

**DOI:** 10.3389/fmicb.2025.1668494

**Published:** 2025-12-02

**Authors:** Caicai Xu, Yong Liu, Junjian Li, Biao Wang, Hong Zhang

**Affiliations:** 1 Institute of Loess Plateau, Shanxi University, Taiyuan, China; 2 College of Environmental and Resource Sciences, Shanxi University, Taiyuan, China

**Keywords:** ecological restoration measures, soil biodiversity, soil network complexity, soil multifunctionality, multiple taxa

## Abstract

**Introduction:**

Intensive opencast coal mining has severely degraded soil ecosystem structure and function. Although ecological reclamation enhances soil biodiversity and multifunctionality (SMF), the underlying mechanisms—particularly how reclamation strategies influence SMF through the α-diversity, β-diversity, and network complexity of soil microbial and eukaryotic communities—remain unclear.

**Methods:**

We employed a space-for-time substitution approach along a 30-year restoration chronosequence at the Antaibao opencast coal mine in northern China. The study included naturally restored grasslands and forests, artificially reclaimed vegetation, and unreclaimed bare land. We quantified SMF as the average Z-score of 19 soil variables related to nutrients, enzyme activities, and microbial biomass. Soil biodiversity (α- and β-diversity) and network complexity of bacterial, archaeal, fungal, and eukaryotic communities were assessed using high-throughput sequencing and topological network analysis.

**Results:**

Ecological reclamation significantly enhanced SMF, with mixed coniferous-broadleaf forests showing the highest level, followed by pure forests and grasslands. The α-diversity of all taxonomic groups and the β-diversity of bacteria and fungi were positively correlated with SMF. Artificially reclaimed sites increased network complexity in bacterial and archaeal communities but reduced it in eukaryotes. Random Forest and multiple regression analyses identified bacterial and fungal β-diversity as the dominant predictors of SMF recovery, followed by the network complexity of bacteria, archaea, and eukaryotes.

**Discussion:**

Our findings demonstrate that reclamation strategy influences SMF through shifts in multidimensional soil biodiversity and network architecture. The results underscore the importance of integrating multi-taxon and multi-dimensional attributes—such as community composition and co-occurrence networks—to fully elucidate how soil communities drive ecosystem multifunctionality during restoration.

## 1 Introduction

Soil biological communities, characterized by their high diversity and complexity, are the cornerstone of soil system functionality (Wang et al., 2023; Chen et al., 2024). Many studies have confirmed that soil microbial diversity plays an indispensable role in sustaining the stability of soil ecosystem functioning (Banerjee et al., 2019; van der Heijden and Hartmann, 2016; Liang et al., 2019; Xu et al., 2024; Du et al., 2025), even the diversity of soil meso-fauna also have predict effects on multiple soil functionality (Kou et al., 2021). Therefore, the multiple soil biological groups should be explicitly considered when examining the driving mechanisms on multiple soil functionality in reclaimed ecosystems (Bastida et al., 2016; Delgado-Baquerizo et al., 2016; Jiao et al., 2022; Lefcheck et al., 2015; Xu et al., 2025).

The previous studies mostly focused on the relationship between single soil (biological) group and single soil functional in artificially reclaimed ecosystems (Xiao et al., 2019; Yan et al., 2020, Guan et al., 2020). A study conducted at the Ramagundam opencast coal mine in India reveals that soil bacteria diversity, acting as the primary catalysts of soil material cycling, are indispensable in facilitating the carbon and nitrogen metabolic processes (Akala and Lal, 2001; Ahirwal et al., 2017a,b). Similarly research founded bacterial and fungi diversity are the primary drivers in restoring soil nutrient functions at the Sonepur-Bazari open-cut coal mine, India (Kumar et al., 2015, 2018). In addition, soil protozoa are involved not only in the decomposition and mineralization of soil organic matter, but also in the cycling of soil carbon, nitrogen, and phosphorus, thereby affecting the maintenance of soil functions (Wang et al., 2023). While existing researches largely established links between specific soil biological groups and individual functions, the ecosystem’s capacity to deliver multiple functions simultaneously–soil multifunctionality (SMF)–necessitates a holistic understanding of how multiple biological groups concertedly drive the SMF.

The multiple soil biological groups diversity include not only the Shannon diversity and number of species but also the community composition (Fuhrman, 2009; Faust and Raes, 2012; Hallam and McCutcheon, 2015; Cao et al., 2023). Traditionally, greater emphasis has been placed on species richness (α-diversity). However, a global-scale study revealed that bacterial β-diversity, rather than α-diversity, serves as the best predictor of multifunctionality in arid ecosystems (Delgado-Baquerizo et al., 2016), providing early key evidence supporting the notion that “β-diversity is more important.” Emerging research further indicates that β-diversity is often a stronger indicator of soil ecosystem multifunctionality (Zhang et al., 2025). Specifically, the strength of the association between β-diversity and soil multifunctionality is significantly greater than that of α-diversity for both bacteria and fungi. When microbial communities become homogenized due to dilution, β-diversity declines, directly leading to the deterioration of key functions such as organic matter decomposition and nutrient cycling (Zhang et al., 2025). The reason why the β-diversity of microbial communities serves as a stronger predictive indicator lies in its reflection of functional redundancy, species complementarity, and the potential of the rare biosphere. These characteristics collectively determine the functional robustness and productivity stability of ecosystems when facing environmental changes (Delgado-Baquerizo et al., 2016). This implies that understanding the variation in microbial communities across different locations provides deeper insights into the health status of soil ecosystems than merely knowing the number of species at individual sites.

Additionally, the complex interconnections among different species also fall within the scope of community structure. Recent research has found that the complexity of the soil microbial network, in conjunction with microbial diversity, collectively drives SMF (Jiao et al., 2022). The network analyses combining potential interactions within a specific ecosystem have been increasingly used to understand species associations between biological community members, and to clarify complexity and stability of the ecosystem functionality (Yuan et al., 2021; Zhai et al., 2024; Du et al., 2025). In recent years, co-occurrence network analysis has been widely accepted by microbial ecologists, and confirmed the soil network complexity is an important factor for driving SMF (Chen et al., 2022; Li et al., 2023; Gong et al., 2024). Such as research on the SMF of forests during different woodland use intensity showed microbial network complexity and diversity together drive the SMF (Li et al., 2023). Soil multitrophic network complexity enhances the link between soil biodiversity and SMF in agricultural systems (Jiao et al., 2022). Therefore, investigating the driving effects of multi-group biological community structure on SMF in reclaimed areas, with consideration of the complexity of biological networks, holds practical significance for developing microbial strategies under different ecological reclamation models in semi-arid mining regions.

Our study was conducted at a 30-years reclaimed area of the Antaibao opencast coal mine in Shanxi Province, China, where the goal of reclamation is ecological restoration, focusing on the rehabilitation of a self-sustaining natural ecosystem. We selected plots containing 8 ecological restoration measures: unreclaimed bare land (CK), naturally restored grassland (NG), original topography forest (OTF), artificially reclaimed grassland (AG), artificially reclaimed forest (AF), artificially reclaimed coniferous forest (ACF), artificially reclaimed broadleaved forest (ABF), artificially reclaimed mixed coniferous and broadleaved forest (ACBM). We collected 40 soil samples, and obtained diversity information on soil bacteria, archaea, fungi, eukaryota using high-throughput sequencing of 16S rRNA genes (for soil archaea and bacteria), ITS genes (for soil fungi), and 18S rRNA genes (for soil eukaryota). Soil biological networks were inferred by generating correlation-based co-occurrence networks for each ecological restoration measures. Complexity indexes of networks, reflected by linkage density per taxa. We also obtained data on a set of 19 soil properties to quantified the SMF. Our aim were (a) to assess the SMF related to soil nutrients, soil microbial metabolism, and soil microbial biomass under various ecological restoration measures; (b) to illuminate the responses of soil biomes to ecological reclamation-type changes by exploring the differences of soil α-diversity, β-diversity and network complexity across changing of ecological restoration measures; (c) to explore the driving effects of soil biodiversity and the soil network complexity on the SMF.

## 2 Materials and methods

### 2.1 Field survey and sampling

**FIGURE 1:**
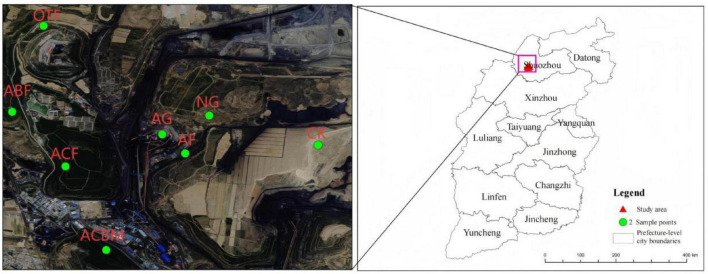
Location of soil samples collection. CK, un-reclaimed bare land; NG, naturally restored grassland; OTF, original topography forest; AG, artificially reclaimed grassland; AF, artificially reclaimed forest; ACF, artificially reclaimed coniferous forest; ABF, artificially reclaimed broadleaved forest; ACBM, artificially reclaimed mixed coniferous-broadleaved forest.

This study was designed on the Pingshuo opencast coal mining area (112°10′–113°30′E, 39°23′–39°37′N), located in the north of Shanxi Province (Figure 1). The region is distinguished by its semiarid temperate continental monsoon climate, which is marked by an average annual precipitation ranging from 428.2 to 449.0 mm. Additionally, the area experiences a moderate climate, with the average annual temperature hovering around 4.8 °C–7.8 °C. The soil type is Calcaric Regosols (WRB-2014) (Bai et al., 1999). Since 1985, the vegetation ecological reclamation has been carried out. Indeed, the ecological environment of the mining area have experienced significant improvement after more than 30 years of consistent vegetation ecological reclamation efforts and the introduction of various plant species.

This study employed a space-for-time substitution chronosequence approach to investigate vegetation and soil development in a reclaimed mining area. Field surveys were conducted in July 2022 across 40 sampling sites representing two restoration patterns (natural restoration and artificial ecological reclamation) and seven specific restoration measures: un-reclaimed bare land (CK), naturally restored grassland (NG), original topography forest (OTF), artificially reclaimed grassland (AG), artificially reclaimed forest (AF), artificially reclaimed coniferous forest (ACF), artificially reclaimed broadleaved forest (ABF), and artificially reclaimed mixed coniferous-broadleaved forest (ACBM). Although reclamation initiatives commenced in different years, all selected sites had reached a relatively stable and mature stage of ecosystem development, with a minimum reclamation duration exceeding 15 years, ensuring comparability across the chronosequence. Soil samples were collected from the 0–20 cm depth layer, where biological activity is most pronounced. Prior to sampling, surface litter was removed, and a five-point sampling method was applied to form composite samples, minimizing spatial bias and enhancing representativeness.

In order to comprehensively assess the soil conditions, two sampling strategies were adopted: On one hand, soil was collected using ring knives to measure soil moisture, a method that accurately reflects the vertical distribution of soil moisture (Zhao et al., 2013). On the other hand, the mixed soil samples were filtered through a 2-mm sieve and then divided into two parts for different treatments. One portion was stored under cold conditions (4 °C) for subsequent DNA extraction and high-throughput sequencing, which would facilitate the analysis of soil biodiversity. The other portion underwent air-drying, intended for determining the basic soil properties.

### 2.2 Soil physico-chemical properties

Soil moisture content was determined by the oven-drying method. To prevent the volatilization of soil organic matter and to maintain the integrity of the samples for subsequent chemical analyses (Robertson, 1999; Schelle et al., 2013), the soil samples were dried at 60 °C for 72 h until a constant weight was achieved. The soil pH was determined by preparing a soil-to-water suspension at a ratio of 1:2.5 (mass to volume) and then measuring it using a glass electrode. For the quantification of soil total carbon (TC) and soil organic carbon (SOC), a spectrophotometric approach was employed following the K_2_Cr_2_O_7_-H_2_SO_4_ oxidation digestion procedure, as outlined by Walkley and Black (1934). After the digestion process with H2SO4, the Kjeldahl method was subsequently applied to measure the total nitrogen (TN) content, a technique validated by Sun et al. (2008). The ammonium nitrogen (NH_4_^+^-N) is determined using the indophenol blue colorimetric method, while the nitrate nitrogen (NO_3_-N) is measured through cadmium reduction followed by spectrophotometry (Searle, 1984; Kempers and Luft, 1988). Total phosphorus (TP) was determined by a colorimetric method using a H_2_SO_4_-HClO_4_ oxidation digestion procedure (Murphy and Riley, 1962). Available phosphorus (AP) was analyzed via colorimetric method after extracted with NaHCO3 (pH = 8.5) (Olsen et al., 1954).

### 2.3 Soil enzyme activity

We determined seven soil enzyme activities related to C-acquiring enzyme, N-acquiring enzyme, and organic P-acquiring enzyme following modified methods (Guan et al., 1986; Tabatabai and Bremner, 1969; Eivazi and Tabatabai, 1988; Steinweg et al., 2012), which including C (α-1,4-glucosidase, β-1,4-glucosidase [BG], β-D-1,4-cellobiohydrolase [CB], and β-1,4-xylanase [XS]) for carbohydrates, N (leucine aminopeptidase, β-N-acetylglucosaminidase [NAG]) for nitrogen, and P (acid phosphatase [AcP]) for phosphorus. The determination method is the traditional spectrophotometry, which is based on the principle that after the enzyme is mixed with the substrate and incubated, it produces a colored product that generates a characteristic peak at a certain wavelength of absorption. Then, a spectrophotometer is used to measure the absorbance values of the set standard and the produced product, thereby determining the amount of enzyme activity (Cui et al., 2018, 2019).

### 2.4 Soil microbial biomass

Soil microbial biomass carbon (MBC), nitrogen (MBN), and phosphorus (MBP) were determined by the fumigation-extraction method (Brookes et al., 1985). Specifically, fresh soil samples were divided into two portions. One portion was exposed to ethanol-free chloroform (CHCl3) vapors for 24 h in a sealed, dark glass desiccator at 25 °C. The other portion served as an un-fumigated control. Following fumigation, the CHCl3 was removed by repeated evacuation. Both the fumigated and control soils were then extracted with 0.5 mL K2SO4 by shaking on a reciprocating shaker. The extracts were subsequently filtered through Whatman No. 42 filter papers. Organic carbon in the extracts was analyzed by potassium dichromate oxidation. Nitrogen was determined as Total N after persulfate oxidation. Phosphorus was measured by the molybdate blue method after persulfate oxidation using a UV-Vis spectrophotometer. The differences in extractable C, N, and P between the fumigated and non-fumigated samples were calculated. MBC, MBN, and MBP were then determined using the following conversion factors:

MBC = EC/KEC, where EC is the extracted C from fumigated soil minus that from non-fumigated soil, and KEC = 0.45 (Brookes et al., 1985).

MBN = EN/KEN, where EN is the extracted N from fumigated soil minus that from non-fumigated soil, and KEN = 0.54 (Brookes et al., 1985).

MBP = EP/KEP, where EP is the extracted P from fumigated soil minus that from non-fumigated soil, and KEP = 0.40 (Brookes et al., 1985).

### 2.5 Soil DNA extraction, PCR amplification and amplicon sequencing

The soil organism DNA was extracted from 0.25 g of soil samples using the E.Z.N.A.^®^ Soil DNA Kit (Omega Bio-tek, Norcross, GA, USA). The final concentration and purity of the extracted DNA were determined using a NanoDrop 2000 UV-Vis Spectrophotometer (Thermo Scientific, Wilmington, DE, USA), and the quality of the DNA was further assessed through 1% agarose gel electrophoresis. PCR amplification of target gene regions utilized the extracted DNA as a template, proceeding as follows: for bacteria, the V3-V4 variable region of the 16S rRNA gene was amplified using upstream primer 338F (5′-ACTCCTACGGGAGGCAGCAG-3′) and downstream primer 806R (5′-GGACTACHVGGGTWTCTAAT-3′) carrying Barcode sequences; for archaea, the V4-V5 region of the 16S rRNA gene was targeted with upstream primer 524F10extF (5′-TGYCAGCCGCCGCGGTAA-3′) and downstream primer Arch958RmodR (5′-YCCGGCGTTGAVTCCAATT-3′); for Fungi, the ITS1 region of the ITS gene was amplified using primers ITS1F (5′-CTTGGTCATTTAGAGGAAGTAA-3′) and ITS2R (5′-GCTGCGTTCTTCATCGATGC-3′); for eukaryota, the 18S rRNA gene was targeted with primers RP841F (5′-GACTAGGGATTGGAGTGG-3′) and Reg1302R (5′-AATTGCAAAGATCTATCCC-3′). Subsequently, PCR amplicons were purified and used to construct sequencing libraries following the standard Illumina protocol. The quality of the libraries was evaluated using an Agilent 2100 Bioanalyzer. Qualified libraries were then sequenced on an Illumina MiSeq platform to generate 2 bp × 300 bp paired-end reads. Raw sequences were subjected to bioinformatic processing with QIIME 2 (v2023.5): primers were trimmed, paired-end reads were merged, and sequences were quality-filtered (quality score ≥ 20), denoised, and checked for chimeras to generate a final set of amplicon sequence variants (ASVs) (Behnke et al., 2011; Caporaso et al., 2011; Gardes and Bruns, 1993; White et al., 1990).

### 2.6 Soil biodiversity and soil multifunctionality

Soil biological α-diversity indices (observed OTUs and Shannon index) were calculated using Mothur software (V.1.30.2) (Jiao et al., 2022). Soil biological β-diversity indices were quantified through Non-Metric Multidimensional Scaling (NMDS) ordination. Soil multifunctionality (SMF) encompasses soil pH, soil water content and various indicators related to soil nutrient content, microbial metabolic activity, and microbial productivity (Garland et al., 2021; Manning et al., 2018). Key indicators for soil nutrient function encompass total carbon (TC), soil organic carbon (SOC), total nitrogen (TN), ammonium nitrogen (NH_4_^+^-N), nitrate nitrogen (NO_3_-N), total phosphorus (TP), and available phosphorus (AP). Microbial metabolic function is characterized by the enzymes activities that are associated with soil carbon, nitrogen and phosphorus cycling, including α-1,4-glucosidase, β-1,4-glucosidase, β-D-1,4-cellobiohydrolase, β-1,4-xylanase, leucine aminopeptidase, β-N-acetylglucosaminidase, and acid phosphatase. Microbial Productivity refers to the soil potential to support and maintain a productive microbial community. Indicators of microbial productivity are soil microbial biomass carbon (MBC), nitrogen (MBN), and phosphorus (MBP), which represent the immediate bioavailable pools of these elements that can be rapidly cycled by microorganisms (Garland et al., 2021; Hu et al., 2021; Manning et al., 2018; Sardans and Penuelas, 2015).

The comprehensive soil multifunctionality (SMF) index was quantified through the integration of 19 biochemical indicators representing four functional domains. The calculation followed a standardized procedure: (1) Individual measurements were converted to Z-scores to normalize scaling differences; (2) Functional dimension indices were computed as arithmetic means of relevant Z-scores: soil nutrient index (SN) from TC, SOC, TN, NH_4_^+^-N, NO_3_-N, TP, AP; soil microbial metabolism index (SMM) from α-1,4-glucosidase, β-1,4-glucosidase, β-D-cellobiohydrolase, β-1,4-xylanase, leucine aminopeptidase, β-N-acetylglucosaminidase, acid phosphatase; soil microbial productivity (SMP) from MBC, MBN, MBP; (3) The integrated SMF index was derived by averaging Z-scores of all 19 indicators plus soil pH and moisture content. This methodology ensures dimensional integration while maintaining functional weighting transparency.

### 2.7 Soil biological network analysis

The co-occurrence networks were constructed based on the OTU abundance table. Prior to construction, OTUs with a relative abundance lower than 0.01% across all samples were removed to minimize the influence of potential sequencing errors and rare species. Additionally, only OTUs that were present in more than 50% of the samples within each group were retained to ensure the robustness of the correlation calculations. This study utilized the “igraph” package in R software, based on Operational Taxonomic Units (OTUs) data from bacteria, archaea, fungi, and eukaryotes to calculate a correlation matrix with the aim of investigating the interrelationships among microbial communities (Csardi and Nepusz, 2006). By setting a threshold (Spearman’s absolute correlation coefficient |r| > 0.8 and *P*-value < 0.05), strong correlation relationships were identified, resulting in datasets of nodes (representing OTUs) and edges (representing correlations between OTUs). Subsequently, these node and edge datasets were imported into Gephi for computing network topological properties and visualizing the complex network of soil biome interactions (Chen et al., 2022). The network complexity were calculated using standardized topological features of each network, including the number of edges, the notes, the average degree and graph density (Jiao et al., 2022). The average linkage density, which represents the average number of links per node in the network and is defined as *L/N*, where L is the total number of links and N is the total number of nodes (taxa).

### 2.8 Statistical analyses

We examined the differences in soil biological diversity, network complexity, and soil multifunctionality among different ecological restoration measures using ANOVA. Following this, Tukey’s HSD test was employed to pinpoint precisely which ecological reclamation strategies exhibit significant differences (with *P* < 0.05). We investigated the correlations among soil biodiversity, network complexity, and indicators related to soil multifunctionality using Spearman’s correlation analysis. We employed Generalized Additive Models (GAMs) to investigate the relationship between network complexity and soil multifunctionality. Furthermore, employing the “randomForest” package in R programming language, we adopted a random forest model to predict and evaluate the importance of soil biodiversity and network complexity in relation to soil multifunctionality.

We utilized the “MuMIn” package in R to conduct a multiple regression analysis, assessing the impact of soil biome factors on soil multifunctionality. This involved calculating the standardized regression coefficients (R^2^) for each explanatory variable in the model and examining their statistical significance. Additionally, we employed the “rdacca.hp” package in R to analyze the relative importance of the explanatory variables, referring to their variance explanation rates, which denote the proportion of the explained R^2^ attributed to each variable. This methodology offers an exhaustive and nuanced perspective on how rehabilitation practices mold the soil multifunctionality dynamics.

## 3 Results

### 3.1 Soil properties and soil multifunctionality

**FIGURE 2:**
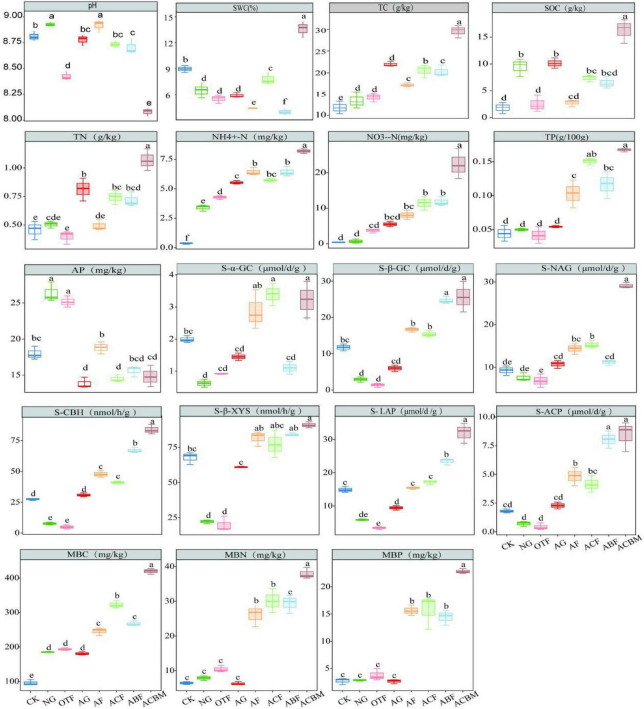
Responses of soil properties to ecological reclamation-type changes. Bars sharing the same lowercase letter are not significantly different (*p* > 0.05) as determined by one-way ANOVA followed by Tukey’s HSD *post-hoc* test. Error bars represent standard deviation.

**FIGURE 3:**
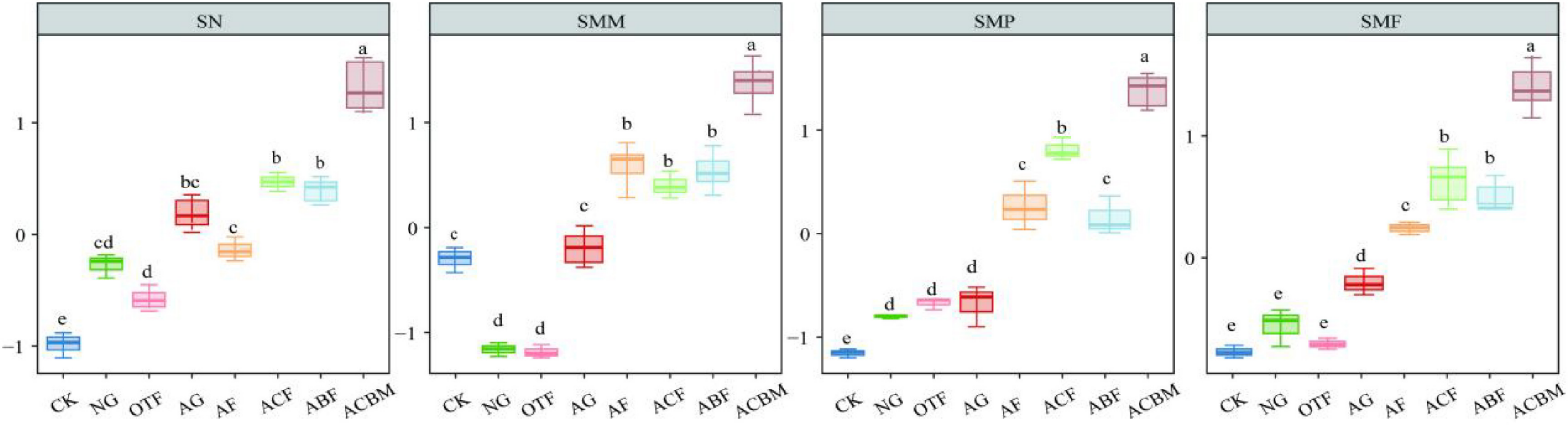
Responses of soil multifunctionality (SMF) to ecological reclamation-type changes. Bars sharing the same lowercase letter are not significantly different (*p* > 0.05) as determined by one-way ANOVA followed by Tukey’s HSD *post-hoc* test. Error bars represent standard deviation.

Artificially reclaimed mixed forests (ACBM) consistently demonstrated superior multifunctionality, showing significantly elevated levels of key soil nutrients (TC, SOC, TN, NH_4_^+^-N, NO_3_-N) and enhanced activities of multiple enzymes (α-GC, S-β-GC, S-CBH, S-LAP, S-ACP) (Figure 2). Furthermore, ACBM supported the highest microbial biomass (MBC, MBN, MBP) among all restoration types. Soil multifunctionality (SMF) was significantly enhanced by ecological reclamation, with reclaimed areas exhibiting notably higher soil nutrient content (SN), microbial metabolic potential (SMM), and microbial productivity (SMP) compared to unrestored bare land (CK) (Figure 3).

While artificial forests (AF) generally outperformed original topography forests (OTF) in most functional aspects, artificial and natural grasslands showed functional complementarity: artificial grasslands (AG) had stronger SMM, whereas natural grasslands (NG) supported higher SMP. Notably, no significant differences in SN were detected between OTF and NG, or between NG and AG, indicating comparable nutrient restoration capacity under these regimes. Overall, artificial reclamation, particularly mixed-species afforestation, most effectively restored comprehensive soil multifunctionality in this degraded mining ecosystem.

### 3.2 Soil biological diversity and composition

**FIGURE 4:**
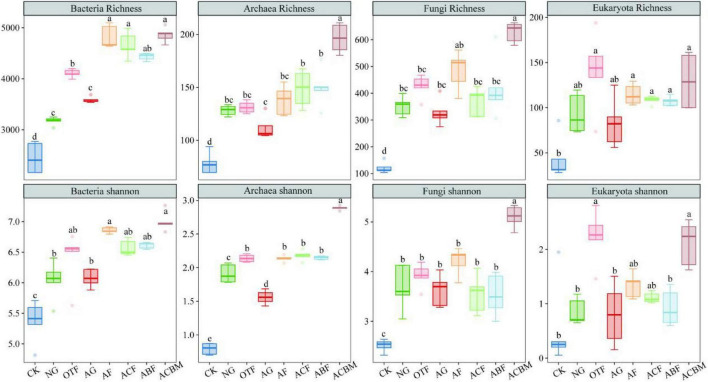
Responses of the soil biological α-diversity to ecological reclamation-type changes. Each plot corresponds to conditions labeled SN, SMM, SMP, and SMF, with varying distributions and distinct letter annotations indicating significant differences.

The effects of ecological reclamation-type changes on soil biological diversity varied with paired ecological restoration measures and microbial communities (Figure 4). The α-diversity of soil biomes in unreclaimed bare land (CK) were significantly lower than that in other plots with vegetation ecological reclamation. The biological α-diversity between NG and AG exhibited no significant differences excepted archaea Shannon. The soil bacterial richness of OTF was significantly higher than NG. However, there were no significant differences in bacterial richness and Shannon diversity between different types of artificially reclaimed forest lands. Similarly, the richness of eukaryotic organisms showed no-significant variation (Figure 4). The Shannon diversity of eukaryotic organisms in ACBM was higher than that in ACF and ABF (Figure 4). The richness of archaea and fungi, as well as the Shannon diversity index of ACBM were significantly higher than in ACF and ABF. The bacterial richness in AF was significantly greater than that in AG. Similarly, the bacterial richness in OTF was significantly greater than in NG. The Shannon diversity index of bacteria in AF showed no significant differences from that in the OTF, and the Shannon diversity index of bacteria in AG was not significantly different from that in NG. Both the richness and Shannon diversity index of archaea in AF were greater than in AG, and the Shannon diversity index of archaea in AF is greater than in NG (Figure 4).

**FIGURE 5:**
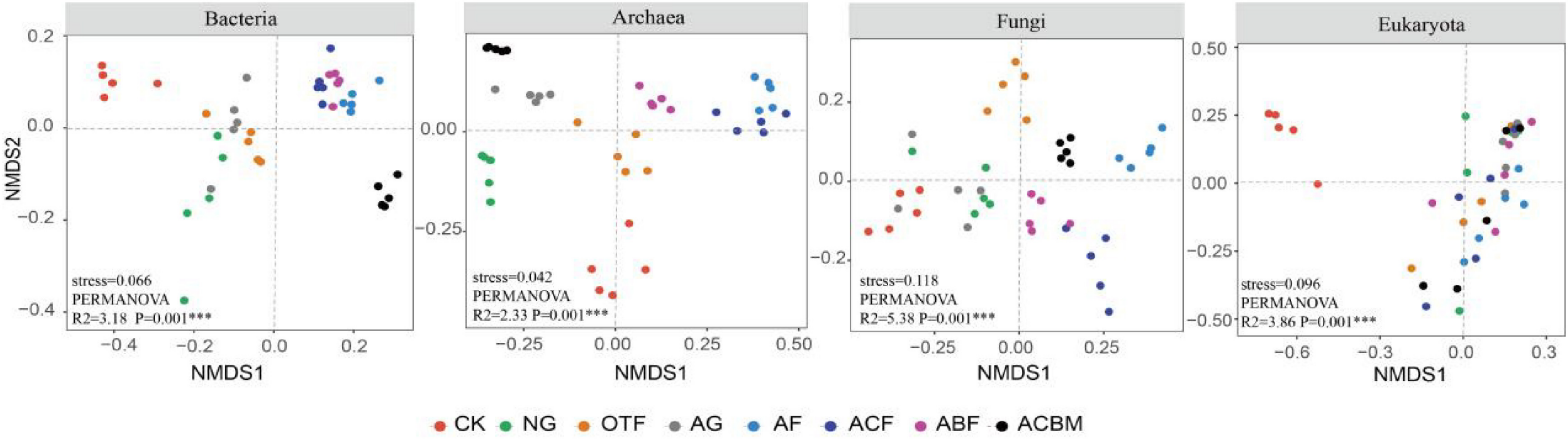
Non-metric multidimensional scaling (NMDS) ordination based on Bray-Curtis dissimilarity shows the variation of bacteria, archaea, fungi and eukaryota communities in different ecological restoration measures.

Non-metric multidimensional scaling (NMDS) analyses revealed systematic divergence in soil microbial community composition across restoration measures, with all domains showing significant separation (*P* = 0.001). The strength of reclamation effects varied substantially among microbial groups: fungi exhibited the highest explanatory variance (R^2^ = 5.38), followed by eukaryotes (R^2^ = 3.86), bacteria (R^2^ = 3.18), and archaea (R^2^ = 2.33). Bare land (CK) consistently formed distinct clusters across all biological domains, demonstrating the universal impact of vegetation establishment on soil microbial assembly. While bacterial and archaeal communities showed convergence between natural and artificial grasslands, fungal and eukaryotic communities maintained stronger differentiation according to restoration approach. Artificial forest types produced more similar community structures across microbial domains, particularly for prokaryotes, whereas eukaryotes exhibited heightened sensitivity to vegetation type. These results demonstrate that ecological reclamation universally reshapes soil microbiomes, with response magnitude following a hierarchical pattern: fungi > eukaryotes > bacteria > archaea, providing critical insights for designing targeted restoration strategies that account for domain-specific microbial responses (Figure 5).

### 3.3 Soil biological network complexity

**FIGURE 6:**
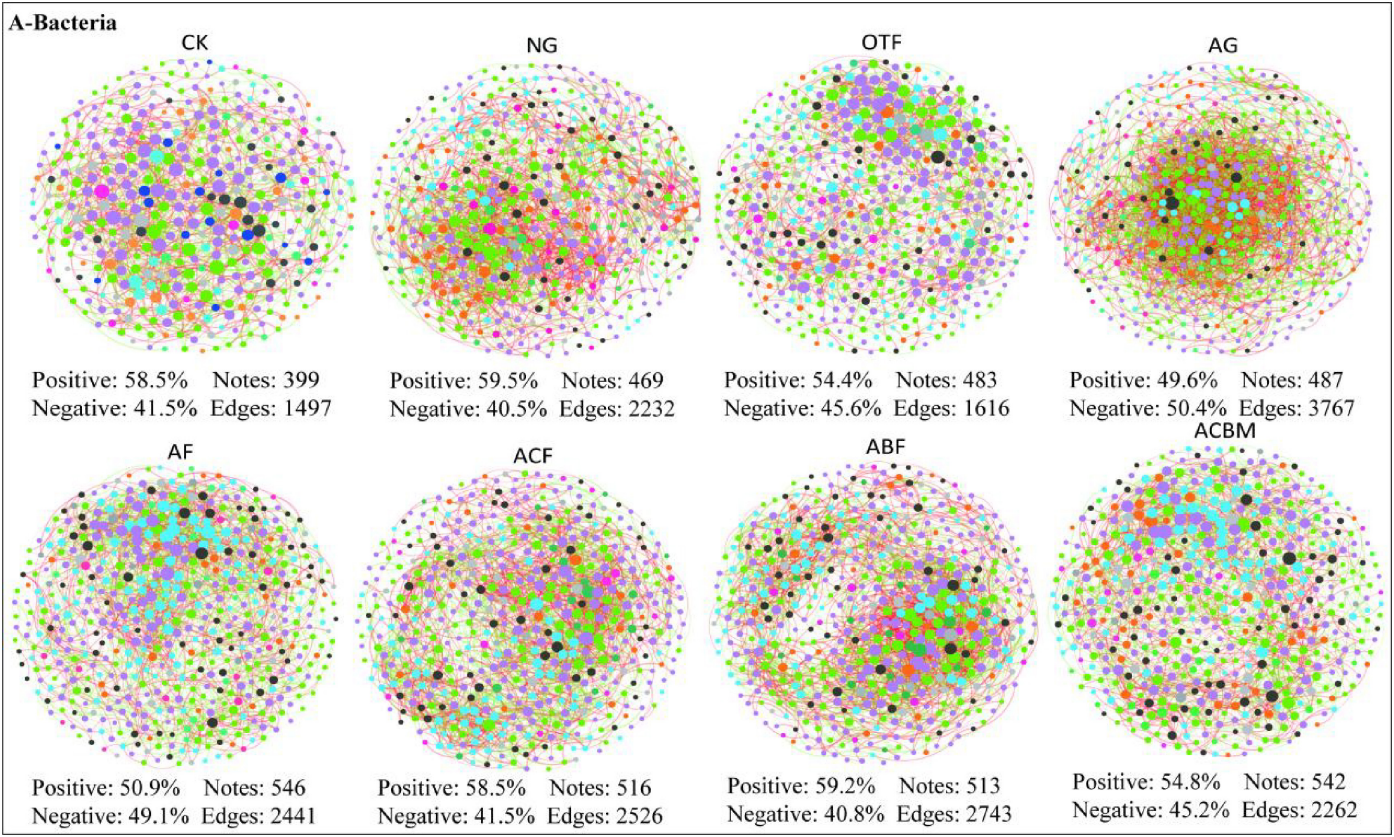
Bacterial co-occurrence networks at the phylum level across different restoration measures. CK, unreclaimed bare land; NG, natural grassland; OTF, original topography forest; AG, artificial grassland; AF, artificial forest; ACF, artificial coniferous forest; ABF, artificial broadleaved forest; ACBM, artificial coniferous-broadleaved mixed forest. Node colors represent different phyla, with node size proportional to relative abundance. Solid lines indicate positive interactions between taxa, while dashed lines represent negative interactions. Network statistics for each site are displayed as: percentage of positive/negative edges, number of nodes, and number of edges.

Network analysis at the bacterial phylum level revealed distinct topological patterns across different reclamation approaches. Artificial grassland (AG) supported the most complex network with the highest number of edges (3767) among all sites, despite having a comparable number of nodes (487) to other vegetated sites (Figure 6). This suggested that grassland restoration promotes particularly dense and interconnected bacterial associations. In contrast, un-reclaimed bare land (CK) maintained the simplest network structure (1497 edges, 399 nodes) (Figure 6), indicating reduced ecological connectivity in degraded soils. The proportion of positive versus negative correlations varied substantially among vegetation types. Artificial grassland (AG) exhibited an almost balanced ratio of positive to negative correlations (49.6%: 50.4%), while other sites showed clear predominance of positive associations (54.4%–59.5% positive) (Figure 6). Artificially reclaimed forests, including coniferous (ACF), broadleaved (ABF), and mixed (ACBM) stands, displayed moderate network sizes (2262–2743 edges) with positive correlation proportions ranging from 54.8% to 59.2% (Figure 6 and Supplementary Table 1). These results demonstrate that reclamation strategy significantly reshapes bacterial interaction networks, with artificial grasslands fostering the most complex network architecture despite more balanced positive-negative link ratios, potentially indicating more stabilized bacterial communities under this restoration approach.

In addition, archaeal networks showed the lower complexity compared to bacteria (maximum 3,725 edges in AF) (Supplementary Figure 1, Supplementary Table 3). Fungal networks demonstrated the highest complexity, particularly in artificial coniferous forest (ACF) with 5,381 edges, while maintaining predominantly positive correlations (51.5%–70.7% positive) (Supplementary Figure 2, Supplementary Table 3). Eukaryotic networks exhibited intermediate complexity, with natural grassland (NG) showing the highest edge density (3,505 edges) among eukaryotic systems. Notably, the proportion of positive correlations varied substantially across domains: archaea (50.6%–56.4%), fungi (50.3%–70.7%), and eukaryota (47.9%–57.3%), with fungal networks in bare land (CK) displaying exceptionally high positive correlation percentages (70.7%) (Supplementary Figures 1–3, Supplementary Tables 2–4). These patterns demonstrate that soil microbial domains respond differentially to reclamation practices, with fungi developing the most complex networks and archaea maintaining relatively simpler architectures, suggesting fundamental differences in ecological organization across microbial kingdoms under vegetation restoration.

### 3.4 Relationships of soil multifunctionality with soil biodiversity and network complexity

**FIGURE 7:**
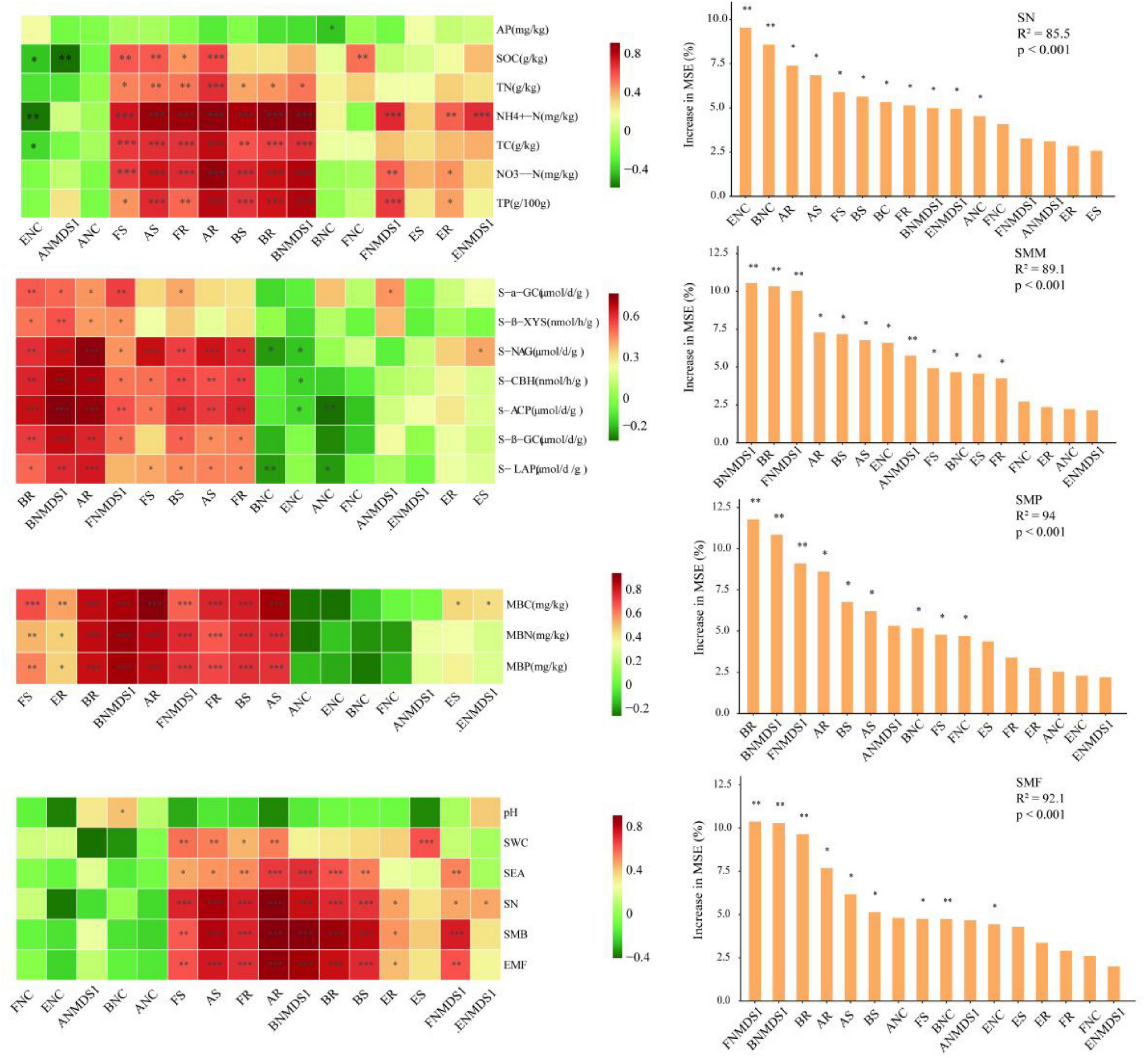
Relationships of soil multifunctionality (SMF) with soil biodiversity and network complexity. The left showed the Spearman correlation analysis of the relationship between soil biotic factors and single soil functions as well as soil multifunctionality; the right showed the importance ranking of soil biotic factors in soil multifunctionality by random forest analysis. *p < 0.05, **p < 0.01, ***p < 0.001

Spearman correlation analysis revealed that the diversity indices of bacteria, archaea, fungi, and eukaryota have significant positive correlations with particular single functions related to SN (*P* < 0.001) (Figure 7). The random forest analysis further demonstrated that the complexity of bacterial and eukaryota networks were crucial and the most significant factors on influencing SN (*P* < 0.01), with the complexity of archaeal networks also had significant impact (*P* < 0.005) (Figure 7). Generalized Additive Model (GAM) analysis revealed that soil microbial network complexity was significantly and positively correlated with soil multifunctionality (SMF), but the strength of this relationship varied substantially across taxonomic groups. Specifically, fungal network complexity emerged as the strongest predictor of SMF (R^2^ = 0.770, *p* < 0.001), followed by eukaryotic (R^2^ = 0.538, *p* = 0.002) and bacterial network complexity (R^2^ = 0.406, *p* = 0.007). In contrast, archaeal network complexity showed no significant association with SMF (Supplementary Figure 4). Correlations between the abundance of bacteria and archaea and single functions associated with microbial metabolic processes were found to be more significant than those of fungi. Moreover, bacterial β-diversity and bacterial abundance emerged as the most important and salient factors for predicting SMM (Figure 7). Regarding to SMP, both Spearman correlation and random forest analyses highlighted the more prominent and significant predictive role played by bacteria compared to the other three biological groups. The diversity indices of bacteria, archaea, and fungi in the soil were positively correlated with SMF, and the community compositions of fungi and bacteria were identified as more pivotal factors influencing SMF compared to other variables (Figure 7).

### 3.5 Soil biological factors contribute to driving soil multifunctionality

**FIGURE 8:**
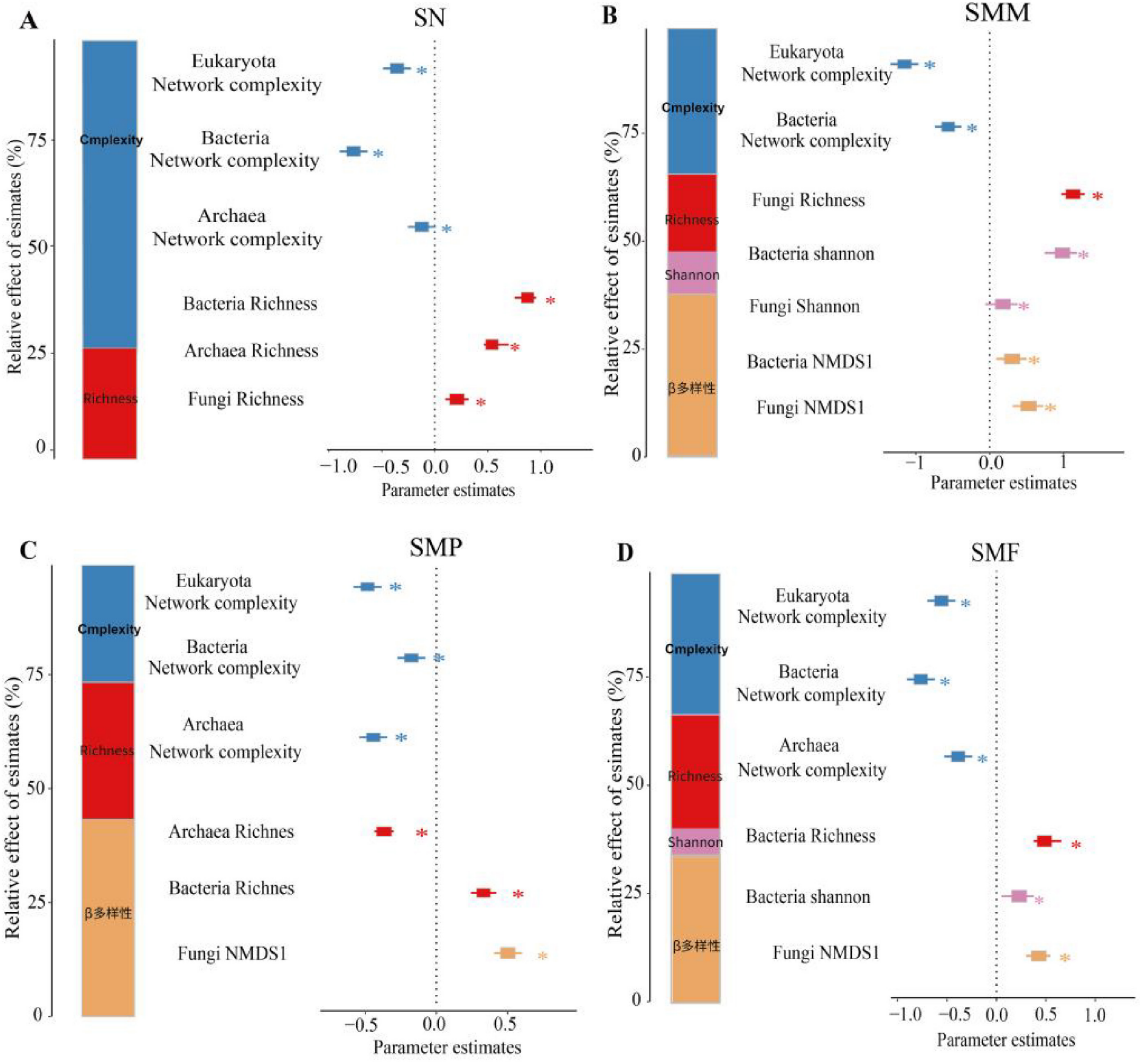
Drivers of soil nutrition (SN) **(A)**, soil microbial metabolism (SMM) **(B)**, soil microbial productivity (SMP) **(C)**, and soil multifunctionality (SMF) **(D)**. Multiple ranking regression reveals the relative importance of the most important predictors of soil functioning (*p < 0.05, **p < 0.01, ***p < 0.001). Bar graphs show the relative importance of each group of predictors, expressed as the percentage of explained variance.

The network complexity and richness played important roles in SN. The explanatory power (contribution proportion) of network complexity was much higher than that of richness, accounting for approximately 75% of the total explanation, demonstrating absolute dominance. Among them, both bacterial and eukaryotic network complexity showed significant negative effects (*p* < 0.05). The contribution of richness to SN accounted for about 25% of the total explanation. Bacterial, archaeal, and fungal richness all showed significant positive effects (Figure 8A).

All biological indicators played important roles in SMM to varying degrees. The explanatory power (contribution proportion) of network complexity accounted for about 35% of the total explanation. Among them, both bacterial and eukaryotic network complexity showed significant negative effects (*p* < 0.05). The contribution of fungal richness to SMM accounted for about 20% of the total explanation, showing a significant positive effect (*p* < 0.05). The contribution of bacterial and fungal Shannon diversity to SMM accounted for about 10% of the total explanation, showing a significant positive effect (*p* < 0.05). The contribution of bacterial and fungal β-diversity to SMM accounted for about 35% of the total explanation, both showing significant positive effects (*p* < 0.05) (Figure 8B).

Network complexity, community richness, and β-diversity all affected SMP. Bacterial, archaeal, and eukaryotic network complexity all had significant negative driving effects on SMP (*p* < 0.05), with a combined contribution proportion of 27%. The contribution of richness to SMP was 28%. Among them, archaeal richness had a significant negative effect (*p* < 0.05), while fungal richness had a significant positive effect (*p* < 0.05). Fungal β-diversity had the highest contribution proportion to SMP (45%), with a significant positive driving effect (*p* < 0.05) (Figure 8C).

All biological indicators played important roles in SMF to varying degrees. Network complexity contributed 31%, mainly driven by significant negative effects from bacterial and eukaryotic network complexity. Bacterial richness contributed 30%, showing a significant positive effect. Bacterial Shannon diversity contributed 5%, showing a significant positive effect. Fungal β-diversity contributed 33%, showing a significant positive effect (Figure 8D).

## 4 Discussion

### 4.1 The ecological restoration measures affect soil multifunctionality and soil biodiversity

Our findings clearly demonstrated the efficacy of ecological restoration in reinstating soil structure and function within open-cast coal mine environments. Our results confirm that soil multifunctionality (SMF) and biodiversity were significantly enhanced following reclamation, with artificially reclaimed mixed forests (ACBM) exhibiting the most comprehensive recovery. This superior multifunctionality in ACBM was underpinned by a significant elevation in key soil nutrients–including total carbon (TC), soil organic carbon (SOC), total nitrogen (TN), and available nitrogen forms (NH_4_^+^-N, NO_3_^+^-N)–coupled with enhanced activities of multiple enzymes related to carbon, nitrogen, and phosphorus cycling (Zhao et al., 2013). This synergy between nutrient accumulation and robust microbial metabolism (reflected in higher microbial biomass MBC, MBN, MBP) creates a positive feedback loop: diverse litter input from mixed forests improves soil structure and provides a heterogeneous resource base, which in turn supports a more diverse and active microbial community (Zhang et al., 2025). This enhanced microbial activity accelerates nutrient mineralization from the litter, further enriching the soil nutrient pool and driving the recovery of overall ecosystem multifunctionality (You et al., 2023). The functional complementarity observed between artificial and natural grasslands, where the former excels in microbial metabolic potential and the latter in productivity, suggests that different reclamation strategies can shape distinct aspects of soil functionality, likely through modulating the soil nutrient status and microbial community composition (Cheng et al., 2023; Liu M.-Y. et al., 2023). Therefore, we conclude that the restoration of a fertile soil nutrient base, catalyzed by appropriate vegetation and microbial communities, is the central mechanism through which ecological reclamation successfully reinstates soil multifunctionality in these degraded landscapes.

In degraded ecosystems, vegetation restoration is a cornerstone for recovering soil multifunctionality (SMF) (You et al., 2023). Our findings demonstrate that successful ecological reclamation significantly enhances the α-diversity of soil bacteria, archaea, fungi, and eukaryotes, all of which showed significant positive correlations with SMF. This improved soil biodiversity acts as a key driver, enhancing soil physicochemical properties and stimulating nutrient cycling, enzyme activities, and microbial metabolism, thereby collectively restoring SMF. Critically, this recovery of soil microbial communities is not spontaneous but is profoundly influenced by the re-established plant communities. The transition to mixed coniferous and broadleaved forests, a common reclamation strategy, enhances litter diversity and accelerates its decomposition (Cheng et al., 2023; Liu S. et al., 2023). This diverse litter input provides a heterogeneous resource base of organic matter and nutrients, which is crucial for supporting a wider range of microbial taxa and potentially increasing microbial β-diversity. Furthermore, the accumulated litter layer modifies the soil micro-environment by regulating temperature and conserving moisture (Soong et al., 2016), creating favorable niches for microbial colonization and activity. Therefore, we propose that plant diversity serves as a foundational driver in this system: it directly enhances litter quality and quantity, which in turn structures the soil microbial assemblages and facilitates the development of complex interactions within them. This cascade of effects–from plant diversity to litter dynamics to microbial community structure and function–ultimately underlies the successful restoration of SMF in these reclaimed lands.

The β-diversity of biological communities, as a key indicator to measure the differences between soil microbial communities, reflects the heterogeneity in the composition of microbial communities across different ecological restoration measures (Delgado-Baquerizo et al., 2017). The β-diversity of bacteria, archaea, and fungi in the soil showed significant differences among ecological restoration measures, but the β-diversity of eukaryotes does not differ significantly between sites. This may be related to the resilience and adaptability of eukaryotic communities. Some studies have found that eukaryotes, such as protists and small metazoans, exhibited a broader ecological niche width and physiological plasticity in reclaimed soils (Wang et al., 2023), allowing them to maintain a relatively stable community structure across different ecological reclamation environments, thus, reducing the differences in β-diversity of eukaryotes. Bacteria, archaea, and fungi exhibited various metabolic rates and activity patterns across different environments, which leading to significant β-diversity among different ecological restoration measures (Köninger et al., 2023). In contrast, eukaryotes possess metabolic pathways that are more adaptable to diverse environments, resulting in less pronounced differences in their β-diversity. The β-diversity of different biological groups reflects the differences in community composition under various habitat conditions. The reasons for these differences include ecological niches, metabolic diversity, species interactions, dispersal abilities, responses to environmental changes, and research methods, among others (Jiao et al., 2022). Future research needs to further explore how these factors affect the β-diversity of different biological communities.

### 4.2 The ecological restoration measures affect soil network complexity

The topological characteristics and complexity indices of microbial co-occurrence networks significantly differed between ecological restoration measures, revealing a domain-specific response: artificial ecological reclamation increased network complexity for bacteria and archaea but decreased it for eukaryotes. This divergence reflects distinct ecological mechanisms operating across trophic levels. For prokaryotes, ecological reclamation-induced shifts in soil properties (e.g., pH and nutrients) enhanced niche differentiation and fostered more specialized interactions, supported by increased resource inputs that promoted cellular activity and cross-feeding opportunities (Jiao et al., 2022). In contrast, eukaryotic networks experienced trophic downgrading, whereby physical disturbance and habitat modification reduced the diversity of higher trophic organisms (e.g., predatory nematodes and protozoa), disrupting predator–prey linkages and simplifying the overall network architecture (Wang et al., 2023).

These domain-level responses are interconnected through cross-trophic cascades. The decline in eukaryotic predators, particularly bacterivorous protozoa, may have relaxed top-down control on bacterial populations, indirectly supporting increased prokaryotic network complexity. Concurrently, compositional shifts in simplified bacterial communities could disrupt co-evolved interactions with specialized eukaryotes, thereby limiting eukaryotic network complexity from the bottom up. Importantly, these local interactions are framed by regional climatic conditions: the semi-arid setting imposes a baseline constraint on network complexity, as documented in arid gradient studies. The observed prokaryotic complexity increase under ecological reclamation thus represents a localized mitigation of climate-driven limitations through improved microhabitat conditions, whereas eukaryotes appear more vulnerable to the combined stresses of local disturbance and regional aridity.

In conclusion, artificial ecological reclamation generates contrasting outcomes for prokaryotic and eukaryotic network complexity–driven by an interplay of bottom-up (environmental filtering, niche differentiation) and top-down (trophic downgrading, cross-domain cascades) processes. These findings underscore the necessity of a multi-domain, food web–aware perspective in evaluating ecological reclamation success. Relying solely on bacterial indicators risks overlooking functional degradation and simplification in higher trophic levels. Future restoration strategies should aim not only to enhance local soil conditions but also to support the functional and structural diversity of the entire soil food web, particularly within water-limited environments.

### 4.3 Soil microbial β-diversity and network complexity jointly drive ecosystem multifunctionality

While the soil biodiversity was widely recognized as the major driver of SMF, most empirical studies have primarily focused on the role of α-diversity. It is important to note that our study, as a snapshot in time, captured the specific stage of restoration process. This temporal limitation is the dynamic successional processes of microbial communities following reclamation were not fully characterized. Despite this constraint, our findings provide robust and novel insights. We found that not only were the α-diversity of bacteria, archaea, fungi, and eukaryotes significantly positively correlated with soil multifunctionality (SMF), but β-diversity also exhibited a significant and often stronger correlation. Most importantly, the β-diversity of bacteria and fungi emerged as the most critical drivers of SMF, demonstrating higher explanatory power than other factors.

Theβ-diversity, which describes the differences in species composition between different sites, is crucial for maintaining SMF by providing spatial functional complementarity. Supporting this, an empirical study along an aridification gradient in the grasslands of Inner Mongolia identified microbial β-diversity as a paramount predictor for sustaining a multitude of ecological processes (Gong et al., 2024). Changes in microbial community composition directly impact the soil environment and key processes like carbon and nitrogen metabolism (Rovira and Vallejo, 2002; Philippot et al., 2013; Jiao et al., 2022). These studies underscore the significance of β-diversity, particularly under stress conditions, where it can serve as a crucial intermediary mitigating adverse effects on SMF. Our results from semi-arid open-pit coal mines confirm the reliability of this concept in reclaimed ecosystems.

Furthermore, the β-diversity provides the template upon which complex soil biological networks are built (Jiao et al., 2022). Network complexity, encompassing interactions such as symbiosis, competition, and predation, is vital for stabilizing ecosystem functions (Delgado-Baquerizo et al., 2020). Our study found that the network complexity of soil bacteria, archaea, and eukaryotes plays an irreplaceable role in driving SMF. This aligns with growing evidence on the critical role of microbial network complexity in maintaining multifunctionality in global ecosystems (Delgado-Baquerizo et al., 2020). A key novelty of our study is that we are the first to explicitly link soil network complexity to SMF in a reclaimed ecosystem, identifying it as a key factor for prediction and regulation.

However, the role of fungal network complexity in driving SMF was diminished in our system. This may be related to the lack of significant differences in fungal α-diversity across different restoration types, potentially leading to simplified interactions. This observation aligns with studies indicating that reduced fungal diversity simplifies network structure, suppresses nutrient availability, and exacerbates degradation (Li et al., 2022). The contrast between bacterial and fungal responses highlights that the recovery of diverse species compositions is a prerequisite for building complex, functional interaction networks (Gong et al., 2024).

In conclusion, our study, while cross-sectional, clearly demonstrates that in reclaimed mine ecosystems, SMF is jointly driven by microbial β-diversity and network complexity. β-diversity provides the spatial variation and functional potential, while network complexity ensures the stability of these functions through species interactions. Future ecological management strategies must therefore focus not only on species richness but also on promoting the development of complex multitrophic networks. We propose that long-term temporal monitoring is essential to unravel the successional trajectories of these biodiversity components and their evolving relationship with SMF, ultimately refining restoration frameworks for predicting and enhancing ecosystem recovery.

## 5 Conclusion

This study establishes that ecological reclamation enhanced the SMF through fundamental restructuring of belowground communities. The mixed forest model (ACBM) demonstrated superior performance in promoting archaeal and fungal diversity while supporting the most complex fungal networks–key determinants of multifunctionality. Crucially, we identified the bacterial and eukaryotic network complexity dominated SN (75% explanation), bacterial β-diversity and abundance primarily predicted SMM, and fungal β-diversity emerged as the strongest predictor of SMP (45% contribution). These findings revealed that different microbial attributes govern distinct functional dimensions. The divergence in network responses to reclamation strategies provides critical insights for restoration management. While bacterial networks thrived in grassland systems, archaeal networks preferred pure forests, and fungal networks achieved maximum complexity in mixed forests. This domain-specific response underscores the necessity of multi-pronged approaches to ecosystem recovery. Furthermore, the consistent advantage of forest systems over grasslands in maintaining eukaryotic network complexity highlights the importance of vegetation structure in supporting higher trophic levels.

Based on these findings, we propose to incorporate specific microbial inoculants (PGPR and AMF) during seedling establishment, with particular emphasis on fungal consortiums given their dominant role in driving multifunctionality. Implement mixed-species planting with optimized broad-leaved to coniferous ratios (approximately 3:2) to create heterogeneous habitats that support diverse microbial networks. Management should focus on maintaining spatial heterogeneity of microbial communities while enhancing key diversity parameters (particularly fungal β-diversity and bacterial richness) that collectively explain over 90% of multifunctionality variance. These strategies collectively provide a validated framework for guiding mining ecosystem restoration by synchronizing aboveground vegetation design with belowground microbial management, ultimately accelerating the recovery of multifunctionality in degraded landscapes.

## Data Availability

The datasets presented in this study can be found in online repositories. The names of the repository/repositories and accession number(s) can be found in the article/Supplementary material.

## References

[B1] Ahirwal J. Maiti S. K. Satyanarayana Reddy M. (2017a). Development of carbon, nitrogen and phosphate stocks of reclaimed coal mine soil within 8 years after forestation with Prosopis juliflflora (Sw.) Dc. *Catena* 156 42–50. 10.1016/j.catena.2017.03.019

[B2] Ahirwal J. Maiti S. K. Singh A. K. (2017b). Changes in ecosystem carbon pool and soil CO2 flflux following post-mine ecological reclamation in dry tropical environment. India. *Sci. Total Environ.* 583 153–162. 10.1016/j.scitotenv.2017.01.043 28095992

[B3] Akala V. A. Lal R. (2001). Soil organic carbon pools and sequestration rates in reclaimed mine soils in Ohio. *J. Environ. Qual.* 30 2098–2104. 10.2134/jeq2001.2098 11790019

[B4] Bai Z. K. Zhao J. K. Li J. C. Wang W. Y. Lu C. E. Ding X. Q. (1999). Ecosystem damage in a large opencast coal mine—Acasestudy on Pingshuo Surface Coal Mine. China. *Acta Ecologica Sinica* 19 870–875. 10.0000/j.1000-0933.20121906870875

[B5] Banerjee S. Walder F. Büchi L. Meyer M. Held A. Y. Gattinger A. (2019). Agricultural intensification reduces microbial network complexity and the abundance of keystone taxa in roots. *ISME J.* 13 1722–1736. 10.1038/s41396-019-0383-2 30850707 PMC6591126

[B6] Bastida F. Torres I. F. Moreno J. L. Baldrian P. Ondono S. Ruiz-Navarro A. (2016). The active microbial diversity drives ecosystem multifunctionality and is physiologically related to carbon availability in Mediterranean semi-arid soils. *Mol. Ecol.* 25 4660–4673. 10.1111/mec.13783 27481114

[B7] Behnke A. Engel M. Christen R. Nebel M. Klein R. R. Stoeck T. (2011). Depicting more accurate pictures of protistan community complexity using pyrosequencing of hypervariable SSU rRNA gene regions. *Environ. Microbiol.* 13 340–349. 10.1111/j.1462-2920.2010.02332.x 21281421

[B8] Brookes P. Landman A. Pruden G. Jenkinson D. (1985). Chloroform fumigation and the release of soil nitrogen: A rapid direct extraction method to measure microbial biomass nitrogen in soil. *Soil Biol Biochem.* 17 837–842. 10.1016/0038-0717(85)90144-0

[B9] Cao T. Luo Y. Shi M. Tian X. Kuzyakov Y. (2023). Microbial interactions for nutrient acquisition in soil: Miners, scavengers, and carriers. *Soil Biol. Biochem.* 188:109215. 10.1016/j.soilbio.2023.109215

[B10] Caporaso J. G. Lauber C. L. Walters W. A. Berg-Lyons D. Lozupone C. A. Turnbaugh P. J. (2011). Global patterns of 16S rRNA diversity at a depth of millions of sequences per sample. *Proc. Natl. Acad. Sci. U. S. A.* 108 4516–4522. 10.1073/pnas.1000080107 20534432 PMC3063599

[B11] Chen B. Pan H. Song X. Yao Y. Qi J. Bai X. (2024). Linking regional species pool size to dispersal–selection relationships in soil fungal communities across terrestrial ecosystems. *Global Ecol. Biogeography* 33:e13876. 10.1111/geb.13876

[B12] Chen W. Wang J. Chen X. Meng Z. Xu R. Duoji D. (2022). Soil microbial network complexity predicts ecosystem function along elevation gradients on the Tibetan Plateau. *Soil Biol. Biochem.* 172:108766. 10.1016/j.soilbio.2022.108766

[B13] Cheng Y.-J. Zhu Y.-F. Ma J. You Y.-N. Dong W.-X. Chen F. (2023). Effects of Plant Restoration On The Functional Genes Of Soil Nitrogen Cycle In Open-Pit Mine Dump. *Chinese J. Soil Sci.* 54 1409–1417. 10.19336/j.cnki.trtb.2022082801

[B14] Csardi G. Nepusz T. (2006). The igraph software package for complex network research. *Inter. J. Complex Syst.* 1695, 1–9. https://api.semanticscholar.org/CorpusID:16923281

[B15] Cui Y. Bing H. Fang L. Jiang M. Shen G. Yu J. (2019). Extracellular enzyme stoichiometry reveals the carbon and phosphorus limitations of microbial metabolisms in the rhizosphere and bulk soils in alpine ecosystems. *Plant Soil* 393 1–14. 10.1007/s11104-019-04159-x

[B16] Cui Y. Fang L. Guo X. Wang X. Zhang Y. Li P. (2018). Ecoenzymatic stoichiometry and microbial nutrient limitation in rhizosphere soil in the arid area of the northern Loess Plateau, China. *Soil Biol. Biochem.* 116 11–21. 10.1016/j.soilbio.2017.09.025

[B17] Delgado-Baquerizo M. Maestre F. T. Reich P. B. Jeffries T. C. Gaitan J. J. Encinar D. (2016). Microbial diversity drives multifunctionality in terrestrial ecosystems. *Nat. Commun.* 7:10541. 10.1038/ncomms10541 26817514 PMC4738359

[B18] Delgado-Baquerizo M. Reich P. B. Trivedi C. Eldridge D. J. Abades S. Alfaro F. D. (2020). Multiple elements of soil biodiversity drive ecosystem functions across biomes. *Nat. Ecol. Evol.* 4 210–220. 10.1038/s41559-019-1084-y 32015427

[B19] Delgado-Baquerizo M. Trivedi P. Trivedi C. Eldridge D. J. Reich P. B. Jeffries T. C. (2017). Microbial richness and composition independently drive soil multifunctionality. *Funct. Ecol.* 31 2330–2343. 10.1111/1365-2435.12924

[B20] Du Y. Yang Y. Wu S. Gao X. He X. Dong S. (2025). Core microbes regulate plant-soil resilience by maintaining network resilience during long-term restoration of alpine grasslands. *Nat. Commun.* 16:3116. 10.1038/s41467-025-58080-2 40169576 PMC11961630

[B21] Eivazi F. Tabatabai M. A. (1988). Glucosidases and galactosidases in soils. *Soil Biol. Biochem.* 20 601–606. 10.1016/0038-0717(88)90141-1

[B22] Faust K. Raes J. (2012). Microbial interactions: From networks to models. *Nat. Rev. Microbiol.* 10 538–550. 10.1038/nrmicro2832 22796884

[B23] Fuhrman J. A. (2009). Microbial community structure and its functional implications. *Nature* 459 193–199. 10.1038/nature08058 19444205

[B24] Gardes M. Bruns T. D. (1993). ITS primers with enhanced specificity for basidiomycetesapplication to the identification of mycorrhizae and rusts. *Mol. Ecol.* 2 113–118. 10.1111/j.1365-294X.1993.tb00005.x 8180733

[B25] Garland G. Banerjee S. Edlinger A. Oliveira E. M. Herzog C. Wittwer R. (2021). A closer look at the functions behind ecosystem multifunctionality: A review. *J. Ecol.* 109 600–613. 10.1111/1365-2745.13511

[B26] Gong X. Jarvie S. Wen J. Su N. Yan Y. Liu Q. (2024). Compared with soil fungal diversity and microbial network complexity, soil bacterial diversity drives soil multifunctionality during the restoration process. *J. Environ. Manag.* 354:120379. 10.1016/j.jenvman.2024.120379 38368806

[B27] Guan S. Zhang D. Zhang Z. (1986). *Soil enzyme and its research methods.* Beijing: Agriculture Press.

[B28] Guan Y. Zhou W. Bai Z. Cao Y. Huang Y. Huang H. (2020). Soil nutrient variations among different land use types after reclamation in the Pingshuo opencast coal mine on the Loess Plateau. China. *Catena* 188:104427. 10.1016/j.catena.2019.104427

[B29] Hallam S. J. McCutcheon J. P. (2015). Microbes don’t play solitaire: How cooperation trumps isolation in the microbial world. *Environ. Microbiol. Rep.* 7 26–28. 10.1111/1758-2229.12248 25721597

[B30] Hu W. G. Ran J. Z. Dong L. W. Du Q. J. Ji M. F. Yao S. R. (2021). Aridity-driven shift in biodiversity-soil multifunctionality relationships. *Nat. Commun.* 12:5350. 10.1038/s41467-021-25641-0 34504089 PMC8429721

[B31] Jiao S. Lu Y. Wei G. (2022). Soil multitrophic network complexity enhances the link between biodiversity and multifunctionality in agricultural systems. *Global Change Biol.* 28 140–153. 10.1111/gcb.15917 34610173

[B32] Kempers A. J. Luft A. G. (1988). Re-examination of the determination of environmental nitrate as nitrite by reduction with hydrazine. *Analyst* 113 1117–1120. 10.1039/an9881301117 3223587

[B33] Köninger J. Ballabio C. Panagos P. Jones A. Schmid M. W. Orgiazzi A. (2023). Ecosystem type drives soil eukaryotic diversity and composition in Europe. *Global Change Biol.* 29 5706–5719. 10.1111/gcb.16871 37449367

[B34] Kou X. Tao Y. Wang S. Wu Z. Wu H. (2021). Soil meso-fauna community composition predicts ecosystem multifunctionality along a coastal-inland gradient of the Bohai Bay. *Land Degradation Dev.* 32 4574–4582. 10.1002/ldr.4053

[B35] Kumar S. Maiti S. K. Chaudhuri S. (2015). Soil development in 2–21 years old coalmine reclaimed spoil with trees: A case study from Sonepur-Bazari opencast project, Raniganj Coalfield. India. *Ecol. Eng.* 84 311–324. 10.1016/j.ecoleng.2015.09.043

[B36] Kumar S. Singh A. K. Ghosh P. (2018). Distribution of soil organic carbon and glomalin related soil protein in reclaimed coal mine-land chronosequence under tropical condition. *Sci. Total Environ.* 625 1341–1350. 10.1016/j.scitotenv.2018.01.061 29996431

[B37] Lefcheck J. S. Byrnes J. E. Isbell F. Gamfeldt L. Griffin J. N. Eisenhauer N. (2015). Biodiversity enhances ecosystem multifunctionality across trophic levels and habitats. *Nat. Commun.* 6:6936. 10.1038/ncomms7936 25907115 PMC4423209

[B38] Li J. Huang X. B. Li S. Tang R. Su J. (2023). Microbial network complexity and diversity together drive the soil ecosystem multifunctionality of forests during different woodland use intensity in dry and wet season. *Forest Ecol. Manag.* 542:121086. 10.1016/j.foreco.2023.121086

[B39] Li J. Li S. Huang X. Tang R. Zhang R. Li C. (2022). Plant diversity and soil properties regulate the microbial community of monsoon evergreen broadleaved forest under different intensities of woodland use. *Sci. Total Environ.* 821:153565. 10.1016/j.scitotenv.2022.153565 35101489

[B40] Liang M. X. Liu X. B. Parker I. M. Johnson D. Zheng Y. Luo S. (2019). Soil microbes drive phylogenetic diversity-productivity relationship in a subtropical forest. *Sci. Adv.* 5:eaax5088. 10.1126/sciadv.aax5088 31681847 PMC6810308

[B41] Liu M.-Y. Chen J.-R. Tian X.-Q. Hu W.-T. Zheng X.-N. Guan X.-F. (2023). Effect of *Eucalyptus robusta* transformation patterns on soil multifunctionality. *J. Shandong Agricultural Univ.* 54 1–12. 10.3969/j.issn.1000-2324.2023.06.016

[B42] Liu S. Plaza C. Ochoa-Hueso R. Trivedi C. Wang J. Trivedi P. (2023). Litter and soil biodiversity jointly drive ecosystem functions. *Global Change Biol.* 29 6276–6285. 10.1111/gcb.16913 37578170

[B43] Manning P. van der Plas F. Soliveres S. Allan E. Maestre F. T. Mace G. (2018). Redefining ecosystem multifunctionality. *Nat. Ecol. Evol.* 2 427–436. 10.1038/s41559-017-0461-7 29453352

[B44] Murphy J. Riley J. P. (1962). A modified single solution method for the determination of phosphate in natural waters. *Anal. Chim. Acta* 27 31–36. 10.1016/S0003-2670(00)88444-5

[B45] Olsen S. R. Cole C. V. Watanabe F. S. Dean L. (1954). *Estimation of available phosphorus in soils by extraction with sodium bicarbonate.* Washington, DC: USDA.

[B46] Philippot L. Spor A. Henault C. Bru D. Bizouard F. Jones C. M. (2013). Loss in microbial diversity affects nitrogen cycling in soil. *ISME J.* 7:1609. 10.1038/ismej.2013.34 23466702 PMC3721106

[B47] Robertson G. P. (ed.) (1999). *Standard soil methods for long-term ecological research.* Oxford: Oxford university press.

[B48] Rovira P. Vallejo V. R. (2002). Labile and recalcitrant pools of carbon and nitrogen in organic matter decomposing at different depths in soil: An acid hydrolysis approach. *Geoderma* 107 10–141. 10.1016/S0016-7061(01)00143

[B49] Sardans J. Penuelas J. (2015). Potassium: A neglected nutrient in global change. *Glob. Ecol. Biogeogr.* 24 261–275. 10.1111/geb.12259 PMC4430815 25983656

[B50] Schelle H. Heise L. Jänicke K. Durner W. (2013). Water retention characteristics of soils over the whole moisture range: A comparison of laboratory methods. *Eur. J. Soil Sci.* 64 814–821. 10.1111/ejss.12108

[B51] Searle P. L. (1984). The Berthelot or indophenol reaction and its use in the analytical chemistry of nitrogen. A review. *Analyst* 109 549–568. 10.1039/an9840900549

[B52] Soong J. L. Vandegehuchte M. L. Horton A. J. Nielsen U. N. Denef K. Shaw E. A. (2016). Soil microarthropods support ecosystem productivity and soil C accrual: Evidence from a litter decomposition study in the tallgrass prairie. *Soil Biol. Biochem.* 92 230–238. 10.1016/j.soilbio.2015.10.014

[B53] Steinweg J. M. Dukes J. S. Wallenstein M. D. (2012). Modeling the effects of temperature and moisture on soil enzyme activity: Linking laboratory assays to continuous field data. *Soil Biol. Biochem.* 55 85–92. 10.1016/j.soilbio.2012.06.015

[B54] Sun Y. N. Bao W. K. Yu M. L. (2008). Determination of total nitrogen content containing fertilizers with semiautomatic determination of nitrogen analyzer. *Soil Fertil. Sci.* 4 69–72. https://api.semanticscholar.org/CorpusID:98873663

[B55] Tabatabai M. A. Bremner J. M. (1969). Use of p -nitrophenyl phosphate for assay of soil phosphatase activity. *Soil Biol. Biochem.* 1 301–307. 10.1016/0038-0717(69)90012-1

[B56] van der Heijden M. G. A. Hartmann M. (2016). Networking in the plant microbiome. *PLoS Biol.* 14:e1002378. 10.1371/journal.pbio.1002378 26871440 PMC4752285

[B57] Walkley A. J. Black I. A. (1934). An examination of the Degtjareff method for determining soil organic matter, and a proposed modification of the chromic acid titration method. *Soil Sci.* 37 29–38. 10.1097/00010694-193401000-00003

[B58] Wang W. T. Wang R. Niu C. P. Bai Y. Yang X. D. (2023). Soil multitrophic ecological network structure of agroforestry rubber plantation in Xi shuang ban na. *Biodiversity Sci.* 31:22626. 10.17520/biods.2022626

[B59] White T. Bruns T. Lee S. Taylor F. White T. Lee S. H. (1990). “Amplification and direct sequencing of fungal ribosomal RNA genes for phylogenetics,” in *PCR protocols: A guide to methods and applications*, eds Innis M. A. Gelfand D. H. Sninsky J. J. White T. J. (New York, NY: Academic Press).

[B60] Xiao L. Bi Y. Du S. Wang Y. Guo C. (2019). Effects of re-vegetation type and arbuscular mycorrhizal fungal inoculation on soil enzyme activities and microbial biomass in coal mining subsidence areas of Northern China. *CATENA* 177, 202–209. 10.1016/j.catena.2019.02.019

[B61] Xu C. Zhang H. Li J. Liu Y. (2025). Assembly processes of both abundant and rare microbial taxa in response to diverse reclamation patterns in an open-cast coal mining. *Plant Soil* 52, 1–16. 10.1007/s.11104-025-07678-y

[B62] Xu C. Zhang H. Li J. Liu Y. Su C. (2024). Microbial diversity drives soil multifunctionality along ecological reclamation chronosequence in an opencast coal mine. *Land Degradat. Dev.* 35 1985–1999. 10.1002/ldr.5031

[B63] Yan B. Sun L. Li J. Liang C. Wei F. Xue S. (2020). Change in composition and potential functional genes of soil bacterial and fungal communities with secondary succession in *Quercus liaotungensis* forests of the Loess Plateau, western China. *Geoderma* 364:114199. 10.1016/j.geoderma.2020.114199

[B64] You Y.-N. Zhu Y.-F. Chen F. Cheng Y.-J. Dong W.-X. Ma J. (2023). Effects of vegetation types on the potential and pathway of microbial carbon sequestration in reclaimed soil of open-pit mine. *J. Ecol. Rural Environ.* 39 1170–1179. 10.1016/j.apsoil.2020.103813

[B65] Yuan M. M. Guo X. Wu L. W. Zhang Y. Xiao N. J. Ning D. L. (2021). Climate warming enhances microbial network complexity and stability. *Nat. Climate Change* 11 343–U100. 10.1038/s41558-021-00989-9

[B66] Zhai C. Han L. Xiong C. Ge A. Yue X. Li Y. (2024). Soil microbial diversity and network complexity drive the ecosystem multifunctionality of temperate grasslands under changing precipitation. *Sci. Total Environ.* 906:167217. 10.1016/j.scitotenv.2023.167217 37751844

[B67] Zhang H. Y. Bao W. K. Hu B. Hu H. (2023). Effect of vegetation type change on soil microbial carbon use efficiency: A review. *Acta Ecol. Sin.* 43 6878–6888. 10.5846/stxb202204161016

[B68] Zhang L. Lei S. Qian R. Ochoa-Hueso R. Wang X. Wang J. (2025). Plant and microbial β diversities are better predictors of ecosystem functioning than their α diversities, but aridity weakens these associations. *Plant Soil* 512 441–460. 10.1007/s11104-024-07093-9

[B69] Zhao Z. Shahrour I. Bai Z. Fan W. Feng L. Li H. (2013). Soils development in opencast coal mine spoils reclaimed for 1–13 years in the West-Northern Loess Plateau of China. *Eur. J. Soil Biol.* 55 40–46. 10.1016/j.ejsobi.2012.08.006

