# Supplementary material for: Soil biodiversity and network complexity jointly drive soil multifunctionality in an open cast coal mine
Source: Front Microbiol. 2025 Dec 2;16:1668494. doi: 10.3389/fmicb.2025.1668494 (PMC12705575; doi:10.3389/fmicb.2025.1668494)
Supplement: Supplementary file 1 [file Data_Sheet_1.pdf]

Soil biodiversity and network complexity jointly drive soil multifunctionality in an open cast coal mine

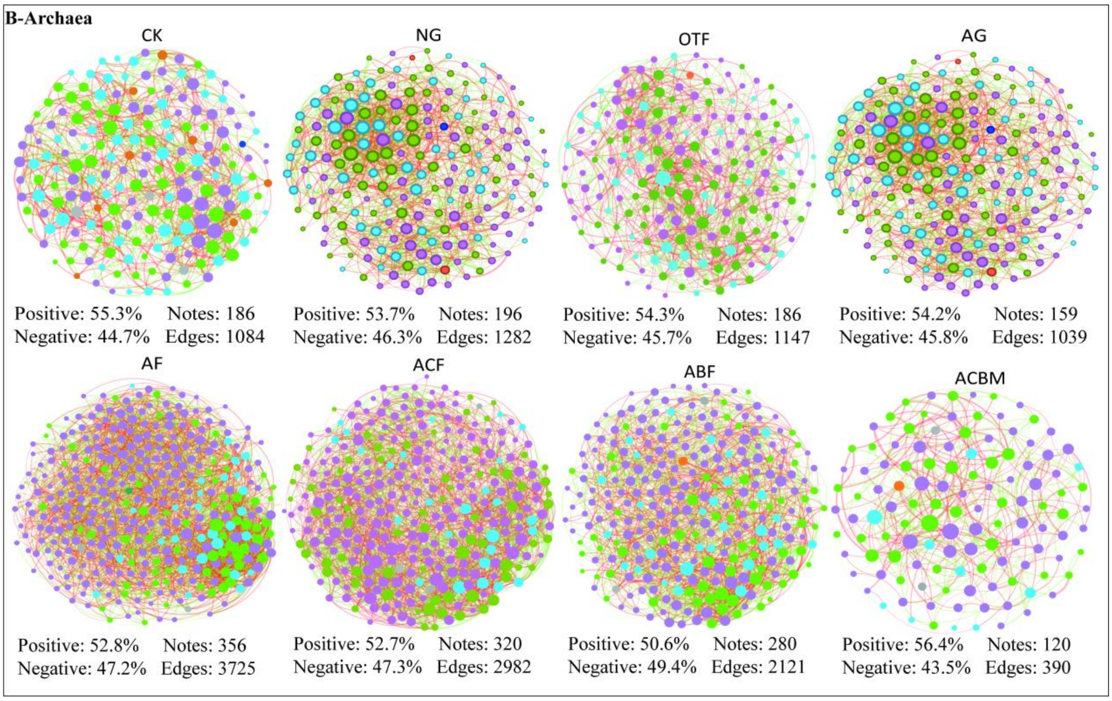

Fig.S1 Archaeal co-occurrence networks at the phylum level across different reclamation types. CK: unreclaimed bare land; NG: natural grassland; OTF: original topography forest; AG: artificial grassland; AF: artificial forest; ACF: artificial coniferous forest; ABF: artificial broadleaved forest; ACBM: artificial coniferous-broadleaved mixed forest. Node colors represent different phyla, with node size proportional to relative abundance. Solid lines indicate positive interactions between taxa, while dashed lines represent negative interactions. Network statistics for each site are displayed as: percentage of positive/negative edges, number of nodes, and number of edges.

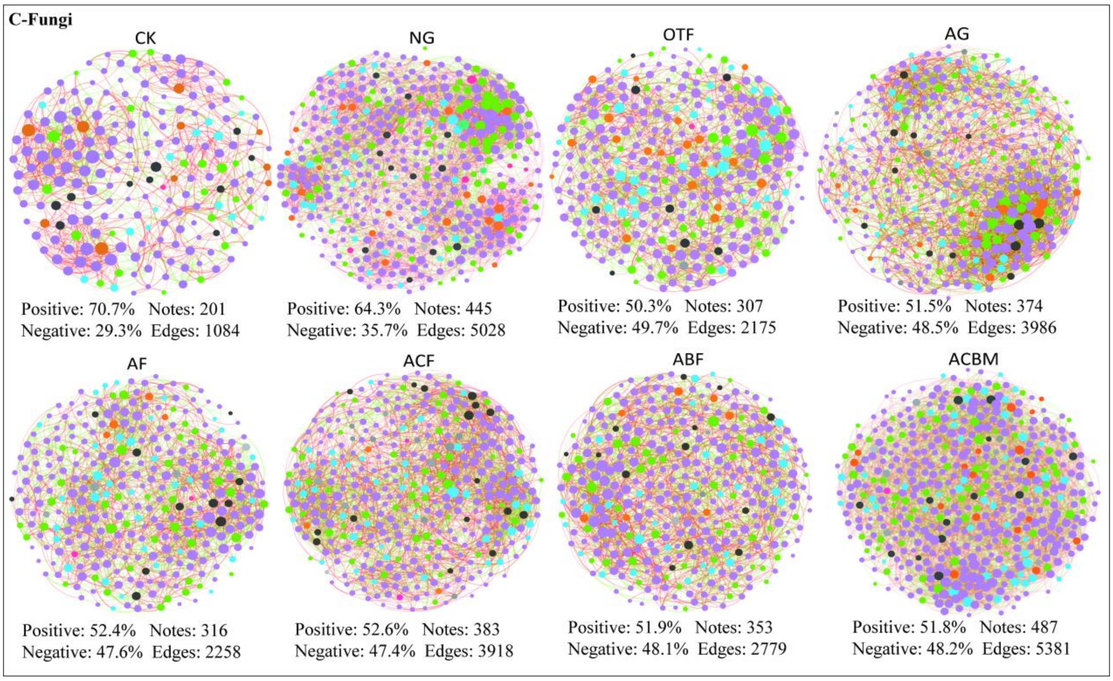

Fig.S2 Fungul co-occurrence networks at the phylum level across different reclamation types. CK: unreclaimed bare land; NG: natural grassland; OTF: original topography forest; AG: artificial grassland; AF: artificial forest; ACF: artificial coniferous forest; ABF: artificial broadleaved forest; ACBM: artificial coniferous-broadleaved mixed forest. Node colors represent different phyla, with node size proportional to relative abundance. Solid lines indicate positive interactions between taxa, while dashed lines represent negative interactions. Network statistics for each site are displayed as: percentage of positive/negative edges, number of nodes, and number of edges.

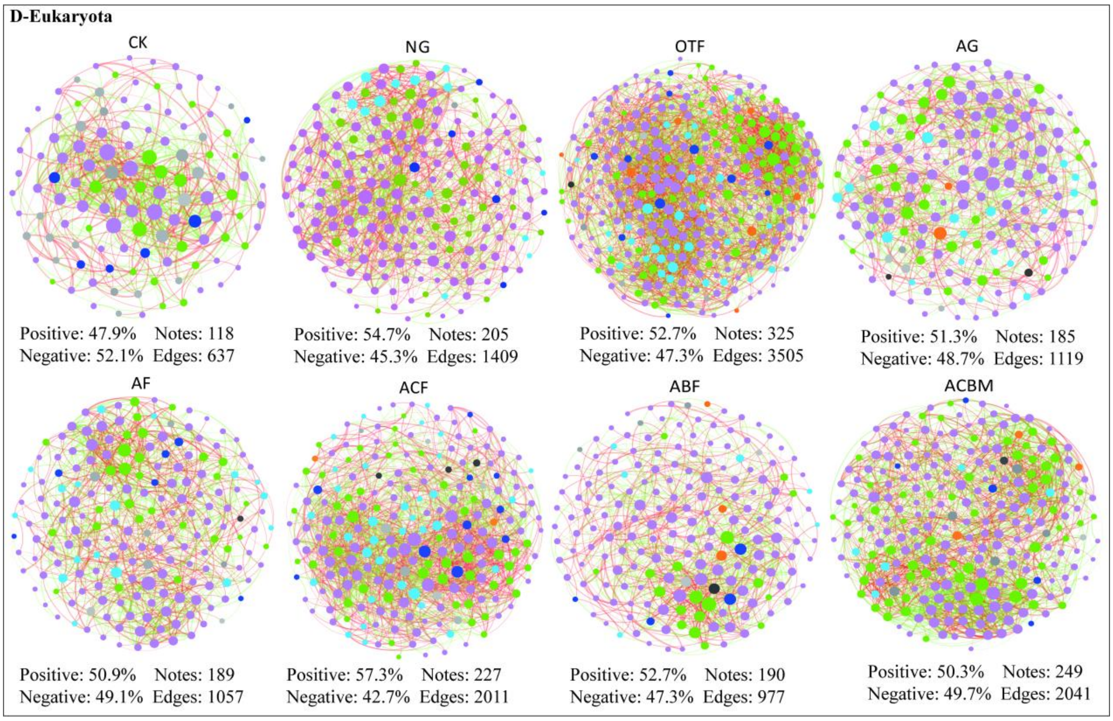

Fig.S3 Eukaryotes co-occurrence networks at the phylum level across different reclamation types. CK: unreclaimed bare land; NG: natural grassland; OTF: original topography forest; AG: artificial grassland; AF: artificial forest; ACF: artificial coniferous forest; ABF: artificial broadleaved forest; ACBM: artificial coniferous-broadleaved mixed forest. Node colors represent different phyla, with node size proportional to relative abundance. Solid lines indicate positive interactions between taxa, while dashed lines represent negative interactions. Network statistics for each site are displayed as: percentage of positive/negative edges, number of nodes, and number of edges.

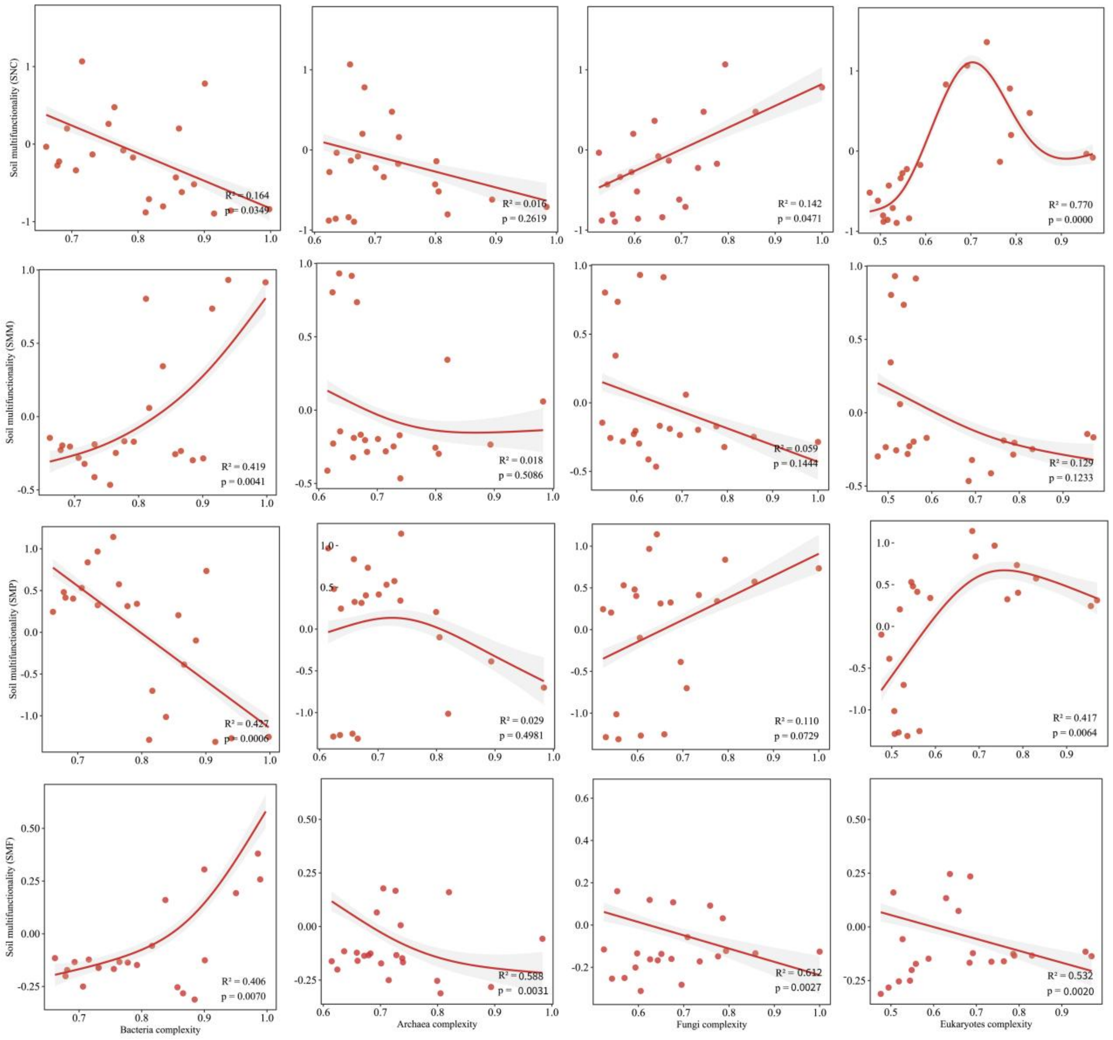

Fig.S4 Generalized Additive Model (GAM) fit for the relationship between soil multifunctionality (SMF) and soil biological network complexity. The fitted smooth function (solid curve) indicates a significant, non-linear dependence of SMF on biological network complexity.

Table S1 Bacteria network properties

Sample	Number of edges	Number of notes	Average degree	Average path length	Diameter	Clustering coefficient	Betweenness centralization
BL	1497	399	7.50376	4.31099	11	0.19857	0.03869
NG	2232	469	9.51812	4.03789	11	0.18997	0.03971
OLP	1616	483	6.69151	4.24643	12	0.17797	0.04247
RG	3767	487	15.47023	3.51797	11	0.24191	0.08405
RCP	2441	546	8.94139	3.96193	10	0.19746	0.02677
PT	2526	516	9.7907	4.04672	11	0.21682	0.04144
RP	2743	513	10.69396	3.92855	10	0.22314	0.03684
PRM	2262	542	8.34686	3.91972	9	0.18122	0.03489

Table S2 Archaea network properties

Sample	Number of edges	Number of notes	Average degree	Average path length	Diameter	Clustering coefficient	Betweenness centralization

BL	1084	186	11.65591	2.68178	6	0.29147	0.03705
NG	1282	196	13.08163	2.60293	5	0.32101	0.02436
OLP	1147	186	12.33333	2.57756	5	0.30224	0.05073
RG	1039	159	13.06918	2.58403	6	0.41674	0.0303
RP	3725	356	20.92697	2.41597	4	0.26769	0.01471
PT	2982	320	18.6375	2.45545	4	0.24348	0.01812
RCP	2121	280	15.15	2.52307	4	0.22701	0.01947
PRM	390	120	6.5	2.92381	6	0.21088	0.06201

Table S3 Fungi network properties

Sample	Number of edges	Number of notes	Average degree	Average path length	Diameter	Clustering coefficient	Betweenness centralization
BL	1084	201	10.78607	3.34627	6	0.42509	0.03677
NG	5028	445	22.59775	2.95741	6	0.42774	0.01271
OLP	2175	307	14.16938	3.04324	6	0.37039	0.01425
RG	3986	374	21.31551	2.94185	6	0.43559	0.01536
RCP	2258	316	14.29114	3.07143	6	0.37116	0.02325
PT	3918	383	20.45953	2.93322	6	0.40314	0.02217
RP	2779	353	15.74504	3.01603	6	0.36579	0.01398
PRM	5381	487	22.09856	2.8592	6	0.36862	0.01109

Table S4 Eukaryota network properties

Sample	Number of edges	Number of vertices	Average degree	Average path length	Diameter	Clustering coefficient	Betweenness centralization
BL	637	118	10.79661	2.81602	7	0.42176	0.05811
NG	1409	205	13.74634	2.56131	5	0.29357	0.03571
OLP	3505	325	21.56923	2.42346	4	0.27878	0.01951
RG	1119	185	12.0973	2.58367	5	0.27484	0.03408
RCP	1057	189	11.18519	2.66548	5	0.25596	0.04585
PT	2011	227	17.71806	2.43456	5	0.29423	0.03335
RP	977	190	10.28421	2.84695	6	0.28242	0.05118
PRM	2041	249	16.39357	2.5207	5	0.29186	0.02205

Table S5 Microbial network correlation

Sample	Bacteria positive	Bacteria negative	Archaea positive	Archaea negative	Fungi positive	Fungi negative	Eukaryota positive	Eukaryota negative
BL	58.5	41.5	55.3	44.7	70.7	29.3	47.9	52.1
NG	59.5	40.5	53.7	46.3	64.3	35.7	54.7	45.3

OLP	54.4	45.6	54.3	45.7	50.3	49.7	52.7	47.3
RG	49.6	50.4	54.2	45.8	51.5	48.5	51.3	48.7
RCP	50.9	49.1	52.8	47.2	52.4	47.6	50.9	49.1
PT	58.5	41.5	52.7	47.3	52.6	47.4	57.3	42.7
RP	59.2	40.8	50.6	49.4	51.9	48.1	52.7	47.3
PRM	54.8	45.2	56.4	43.5	51.8	48.2	50.3	49.7
